# Clinical outcomes of MyoRing implantation in keratoconic eyes by using the Femtosecond laser technology

**Published:** 2015

**Authors:** K Nasrollahi, L Rezaei, M Ghoreishi, A Kashfi, M Mahboubi

**Affiliations:** * Isfahan University of Medical Sciences, Isfahan, Iran; ** Kermanshah University of Medical Sciences, Kermanshah, Iran,; *** Abadan School of Medical Sciences, Abadan, Iran

**Keywords:** MyoRing, keratoconus, femtosecond laser

## Abstract

**Purpose:** To assess the outcomes of the insertion of the MyoRing (DIOPTEX, GMBH, Linz, Austria) by applying the Femtosecond Laser Technology (FLT) method in eyes that have keratoconus problem.

**Methods:** A prospective, nonrandomized, clinical examination was managed. 27 eyes of 15 subjects with stable keratoconus (6 females and 9 males), with lifetimes differing from 14 to 49 years were involved. All subjects have problems about decreased fine-accurate ocular awareness, lens intolerantness or trouble, and also the middle corneal height larger than 350 micrometers. MyoRing additions of about 320 micrometers into height and 2.5 mm into radius were inserted in each subject inside an Intrastromal Corneal Pocket using(ICP) formed with applying FLT. Ocular, refractive errors, corneal shape, and pachymetry changes were assessed throughout a 6-months follow-up period.

**Results:** The average UDVA (uncorrected distance visual acuity) notably increased originating at 1.73 ± 0.53 LogMAR preoperationally to 0.54 ± 0.40 LogMAR post-operationally. The average CDVA (corrected distance ocular awareness) notably increased from 0.59 ± 0.47 LogMAR preoperationally to 0.27 ± 0.16 LogMAR post-operationally. The change in the average UDVA and CDVA was analytically meaningful (P< 0.000). The average reduction in the average keratometry from preoperational to 26 weeks post operational was -6.41 ± 3.62 D. This change was analytically meaningful (P< 0.000). The average lowest and highest keratometry amounts were similarly analytically notable at shorter than 26 weeks preoperatively. A notable increase in UDVA and CDVA was observed 26 weeks following the operation, that was compatible with the notable decrease in sphere and cylinder. Moreover, a notable corneal straightening with an average amount of -6.41 ± 3.62 diopters (D) was determined.

**Conclusion:** MyoRing implantation employing FLT would be a harmless, effective, and predictable method to treat selected subjects of keratoconus, being a helpful choice for the therapy of keratoconus.

## Introduction

One of the non-inflammatory progressive corneal thinning is Keratoconus, characterized by inferior nasal steepening. The corneal thinning induces high regular and irregular astigmatism, often with myopia, creating moderate to a marked deterioration in the state of vision. This disorder is normally bilateral, although one eye may be affected initially [**1**-**3**]. In many of situations, the cornea settles cleared and the refractive fault is handled with stiff contact lenses or spectacles. In advanced keratoconus with corneal opacities and scarring, penetrating keratoplasty (PK) is an accepted surgical plane. In patients who are intolerant to spectacles or rigid contact lens when the cornea remains clear [**1**-**3**].

Corneal designing by entering intrastromal inserts has been purposed and examined as a choice to treatment possibility in corneal keratoconus [**4**]. The application of full-ring inserts has also been purposed as a possible answer for the therapy of erratically formed keratoconic corneas [**5**-**6**].

A new operational choice mentioned to as the “corneal intrastromal implantation system” (CISIS), in which the MyoRing elastic full-ring insert (DIOPTEX GMBH, Austria) is entered into a corneal opening, and has recently been improved and determined to be useful in keratoconus [**7**].

A specified mechanical device improved for CISIS, the Pocket Maker (DIOPTEX GmbH), has been applied to presently for the production of this intrastromal pocket. MyoRing implantation by employing this mechanical method that has been confirmed to be secure also efficient in reducing problems of myopia, corneal ramp, and the corneal top out of centers. [**5**-**8**]. But, it is well recognized that FLT may enable the surgery operator to plan a corneal stromal operation in a planned bottom with a very great level of correctness [**9**], which eludes the possible mistakes of a mechanical operation. In an earlier study of a research group [**4**], it was confirmed that the intrastromal loop portion inserts by utilizing both mechanical and FLT-assisted methods produced alike visual and refractive results. However, a further limited aberometric improvement was begun in eyes with mechanical insertation[**4**].

The existing research assesses the visual, refractive, corneal topography and, pachymetry results following MyoRing insertion in eyes with corneal keratoconus by using the FLT for the creation of the intrastromal pocket required for the complete ring insertion.

The aim of the examination was to prospectively assess the visual outcome from MyoRing implantation by utilizing FLT in the eyes that have keratoconus problem, in an Isfahan for the evaluation of results in another Iranian population.

## Patients and Methods

In this prospective nonrandomized trial research, we assessed eyes that have keratoconus problem, that was treated by the insertion of MyoRing (Dioptex GmbH, Linz, Austria) in a corneal hollow formed by using FLT. The research involved 27 eyes of 15 patients with lifetimes covering from 14 to 49 years. patients. The institutional moral review committee confirmation was achieved for the methods and the principles of the Helsinki Declaration have complied. All the subjects were determined with corneal keratoconus based on the standard models. Keratoconus analysis was established on corneal topography and slit-lamp investigation: the asymmetrical bowtie model including or not including skewed axis and the existence of stromal decrease, the conical projection of the cornea at the apex vogt striae [**10**].

The hardness of Keratoconus was classified based on the Amsler Krumeich arrangement [**11**]. 

- Phase 1: unusual steeping; myopia or caused astigmatism of smaller than 5.00 D, or both; and average middle k indications of smaller than 48.00 D.

- Phase 2: myopia either caused astigmatism ranging 5.00 D extending to 8.00 D, either both; and average middle k indications of smaller than 53.00 D; lack of wound mark; and smallest corneal height of larger than 400 um. 

- Phase 3: myopia either caused astigmatism ranging 8.00 D extending to 10.00 D, either both; average middle k indications of larger than 53.00 D; lack of wound mark; and smallest corneal height of 300 extending to 400 um. 

- Phase 4: no measurable refraction, average middle k indications of larger than 55.00 D; middle corneal wound mark; and smallest corneal height of 200 um.

The inclusion threshold was keratoconus, decreased top reformed ocular awareness, lens intolerance or trouble and central corneal height of larger than 350µm. The exclusion criteria were, pregnancy, active ocular disease (cataract, glaucoma and diabetic retinopathy), past of herpes keratitis, previous ocular surgery, diagnosed autoimmune disease, systematic combinative tissue condition and any previous corneal surgery, concurrent corneal infections, patients with poor compliance, then an informed approval was received from all the subjects. A complete ocular examination including slit lamp examination, fundoscopic examination, manifest refraction, uncorrected distance visual acuity (UDVA), corrected distance visual acuity (CDVA), spherical and cylindrical elements of the obvious refraction, spherical equivalent (SE), keratometry values and corneal thickness, were calculated by pentacam HRS system (Oculus, Optikgerate GmbH, Germany) one month and 6 months later MyoRing insertation. The UCVA and BCVA were collected in decimal scaling and converted into LogMAR for the statiscal analysis.

**Surgical procedures**

Surgical methods were conducted under local anesthesia (Tetracaine 0.5% ophthalmic drops, Darou Pakhsh Phama Co., Iran) by the same expert surgery operator (M. Gh).

All MyoRings were implanted into ICPs. Pocket reaction was performed with femtolaser (Zeimer Ophthalmic System Group, Port, Switzerland).

The pocket diameter was of 8mm with 300µ depth. Pocket entrance was selected at supratemporal position and the size was 5 or 6 mm based on the MyoRing size. The ring was inserted into the opening and was centered based on the pupil with mild decentration according to cone center.

The antibiotic eye drop (Ciplex, Ciprofloxacin HCl 0.3% Sinadarou, Tehran, Iran) was instilled and bandage contact lens was inserted.

**Postoperative management**

All the patients were given topical Ciplex eye drops (Ciprofloxacin HCL 0.30% Sina Daro, Tehran, Iran) and Betasonate eye drops (Betamethadone 0.1%, Sina Daro, Tehran, Iran) four times a day for seven days. 

The postoperative visit was programmed for the initial postoperative day, the initial week, 1 month and 6 months later operation. The first and seventh day after the procedure, the patients were examined for survey epithelial healing, postoperative infection, MyoRing location, and corneal completeness. In the left postoperative interviews, the similarly clinical investigations as preoperatively were completed.

**Statistical analysis**

Information was examined by using SPSS software (v 18; SPSS, Inc., Chicago, IL). Analytical comparisons of preoperational and also post operational values were conducted by applying t-student examination for UCVA, BCVA, average refractive SE, and average K-values. Statistical data are presented as average ± SD. The changes in data were considered analytically notable when the P amount was lesser than 0.05. 

## Results

MyoRing segments were strongly rooted in all eyes without any intraoperative complication. All the subjects finished the 6-months post operational follow-up. A total of 27 eyes of 15 patients were included. The average lifetime of the 6 females (40%) and 9 males (60%) was 28.35 ± 8.29 years (ranging between 14 and 49 years). The average central corneal height was 422.42 ± 36.96 preoperatively and postoperatively it was 445.08 ± 28.00 (p=0.020). There was a statistically meaningful growth in all parameters from preoperatively to postoperatively (**Table 1**).

**Visual awareness**

Unreformed ocular awareness, best-reformed distance pre-operationally and 26 weeks post-operationally procedures are given in **Table 1**. The average UDVA notably improved originating at 1.73 ± 0.53 LogMAR post-operationally to 0.54 ± 0.40 MAR post-operationally. The average CDVA notably increased originating at 0.59 ± 0.47 pre-operationally to 0.27 ± 0.16 post-operationally. The change in the average UDVA and also CDVA during the follow-up period was statistically notable (P< 0.000).

Preoperatively, the UDCA, was 0.1 (20/ 200) or worse in 23 (0.85%) eyes and postoperatively, it was 0.5 (20/ 40) or superior in 9 (33%) eyes.

**Table 1:** The means and standard deviations for all data (preoperative and postoperative)

Parameter	Preoperative	Postoperative	Mean Difference*	P value**
UDVA (LogMAR)	1.73 ± 0.53	0.54 ± 0.40	-1.19 ± 0.59	0.000
CDVA (LogMAR)	0.59 ± 0.47	0.27 ± 0.16	-0.32 ± 0.49	0.002
Sphere (D)	-7.86 ± 3.70	-0.94 ± 2.50	6.92 ± 3.67	0.000
Cylinder (D)	-4.25 ± 2.39	-1.87 ± 1.24	2.55 ± 2.00	0.000
SE (D)	-9.99 ± 3.83	-1.88 ± 2.83	8.11 ± 3.48	0.000
kMIN (D)	49.78 ± 3.57	44.78 ± 2.99	-5.08 ± 3.58	0.000
K MAX (D)	53.81 ± 4.15	47.60 ± 3.58	-6.42 ± 4.24	0.000
Average k (D)	51.97 ± 3.43	45.24 ± 2.61	-6.41 ± 3.62	0.000
UDVA= uncorrected distance visual acuity; CDVA= corrected distance visual acuity; SE= spherical equivalent; K= keratometry				
* Mean Difference from the preoperative period to 6-months postoperatively				
**P amount for the separation within Preoperational and 26 weeks post operational amounts				

The average effectiveness indicator (proportion of postoperational UDVA and preoperational CDVA) was 1.36 ± 1.51 (range 0.2 to 8). 

**Fig. 1:**
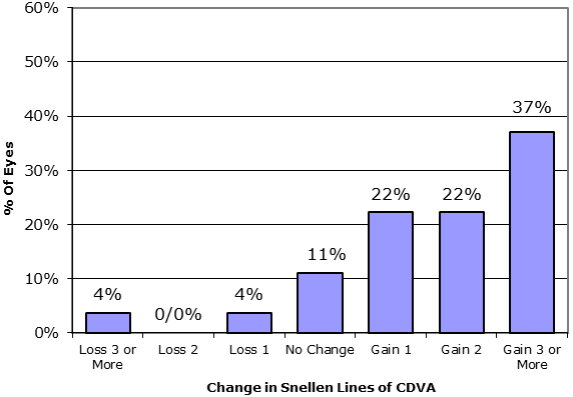
Safety distance ocular awareness graph (% of eyes with gain/ less in snellen lines), at 6 months visit.

As presented in **Fig. 1**, the safety graph, of the 27 eyes, 2 eyes (8%) lost lines of CDVA and 16 eyes (59%) acquired 2 lines or extra at the final follow-up (mean safety index was 2.01 ± 1.66).

Preoperative and 6-months postoperative refraction values are presented in **Table 1**. The average Sphere equivalent (SE) refraction reduced notably from -9.99 ± 3.83 D preoperatively to -1.88 ± 2.83 D at 26 weeks. The average decrease was 8.11 ± 3.48 D. All variations in the average SE, sphere, and cylinder refraction was analytically notable [P< 0.00].

**Corneal curvature (K min, K max)**

The average reduction in the average keratometry from the preoperative period to 6 months postoperatively was -6.41 ± 3.62 D. This change was statistically significant (P< 0.00). The average smallest and largest keratometry amounts were also analytically significant at shorter than 26 weeks preoperatively (**Table 1**).

Patients were divided into 3 collections according to their preoperational keratometry (collection 1, keratometry ≤48 D); collection 2, keratometry 48-53 D; group 3, keratometry >53 D) and analyzed the outcomes of the 3 collections (**Table 2**,**3**).

**Table 2** displays the average ocular awareness and **Table 3** the average refractive results covering the time based on preoperational keratometry. In the eyes with a preoperational keratometry of 48.0 D either smaller and of 48.0 to 53.0 D, the development in UDVA and CDVA and decrease in SE and refractive cylinder were analytically meaningful (**Table 2**,**3**).

In eyes with a preoperational keratometry of 53.0 D either higher, the development in UDVA and decrease in se were analytically notable; and, the variations in CDVA were not significant.

**Table 2:** average visual awareness results by preoperative average keratometry

Preop. Keratometry (n=27)	Mean UDVA ± SD		p. amount	Average CDVA ± SD		p. value
	Preop.	Postop.		Preop.	Postop.	
≤48 (5)	1.80 ± 0.45	0.38 ± 0.23	0.001	0.50 ± 0.08	0.49 ± 0.32	0.98
48-53 (10)	1.49 ± 0.67	0.39 ± 0.30	0.000	0.58 ± 0.57	0.26 ± 0.21	0.094
>53 (12)	1.89 ± 0.37	0.68 ± 0.47	0.000	0.64 ± 0.49	0.28 ± 0.13	0.051

Although we had good results in UDVA outcomes in 3 groups, the improvements in CDVA were more significant in group 1 [keratometry ≤48 D] compared to the 2 other groups.

**Table 3:** average refractive results by preoperative average keratometry

Preop. keratometry	Mean SE ± SD		p. value	Mean cylinder ± SD		p. value
	Preop.	Postop.		Preop.	Postop.	
≤48	-10.5 ± 5.46	-3.57 ± 5.15	0.001	-6.10 ± 1.77	-2.25 ± 1.66	0.002
48-53	-8.22 ± 2.28	-1.21 ± 1.09	0.000	-3.50 ± 1.65	-1.63 ± 1.03	0.010
>53	-11.24 ± 3.87	-1.72 ± 2.58	0.000	-4.40 ± 2.60	-1.87 ± 1.32	0.003

In spite of the notable development in the visual awareness outcomes for all cases, there was not notable development between them for eyes with the central corneal thickness of less than 400 µm (**Table 4**).

**Table 4:** Average ocular awareness results by preoperative average central corneal thickness

Preop. central corneal thickness	Mean UDVA ± SD		P. value	Mean CDVA ± SD		P. value
	Preop.	Postop.		Preop.	Postop.	
<400	1.35 ± 0.75	0.43 ± 0.25	0.094	0.29 ± 0.15	0.25 ± 0.13	0.47
≥400	2.00 ± 0.00	0.47 ± 0.27	0.000	0.61 ± 0.50	0.24 ± 0.14	0.012

## Complications

There were no severe postoperative complications. Mild glare was reported in many of the subjects, particularly in the beginning postoperative time. No MyoRing was removed for side effects or complications.

## Discussion

Keratoconus is an ectatic corneal dysfunction with advancing steepening and corneal sliming, particularly in the secondary section of the cornea. By implanting intrastromal inserts, corneal remodeling can enhance the visual awareness, modifying the deflection of the ectatic cornea [**4**]. Unfinished rings accessible on the market are Intacs, Ferrara ring, and Keraring. The implanting of an entire intrastromal loop, MyoRing (Dioptex GmbH, Austria), is an alternative method, that has been harmless and useful in the earlier investigations in the therapy of keratoconus [**5**-**7**,**11**-**13**].

The principal benefits of a perfect loop are simple insertions, good centeredness, and the postoperative opportunity of changing the ring position, if necessary [**6**].

A mechanical equipment was particularly generated for the making of this intrastromal pocket [**9**].

F technologyLT may allow a surgery operator to perform a corneal stromal operation at a planned depth with a very great level of correctness, thus bypassing the possible defects of a mechanical operation that is based on the surgeon's manual skills [**14**,**15**].

The present research examined the visual, refractive, pachymetry and keratometry results after MyoRing insertion in eyes with Keratoconus by using the FLT for the production of intrastromal pocket in an Iranian population.

In this present study, at one-month later operation, a statistically meaningful decrease in myopia and cylinder was recognized, without any notable variations throughout the resting follow-up. At 6 months, the mean decrease in sphere was 6.92 ± 3.67 D and the average refractive in refractive cylinder was 2.55 ± 2.00 D. These degrees of refractive variation were compatible with those earlier published after MyoRing insertion [**8**-**9**]. 

In Alio et al. study, a whole of 12 eyes of 11 subjects with lifetimes extending from 17 to 50 years was involved. All subjects showed with decreased CDVA, lens intolerances or trouble, and middle corneal height larger than 350 micrometers. MyoRing added about 280 micrometers in height and also 2.5 millimeters in radius were inserted in any subject within an ICP generated by means of FLT. A notable development in UDVA was recognized which was compatible with the notable decrease in sphere and cylinder. There was an average variation in the sphere of 4.62 D and an average variation in the cylinder of 4.47 D [**11**].

As supposed, the notable decrease of refractive errors obtained with MyoRing inserts in our research was associated with a notable increase of UCVA. The average UDVA notably improved originating at 1.73 ± 0.53 LogMAR pre-operationally to 0.54 ± 0.40LogMAR post-operationally. The average CDVA notably improved originating at 0.59 ± 0.47 LogMAR preoperationally to 0.27 ± 0.16 LogMAR post-operationally. The change in the mean UDVA and also CDVA throughout the follow-up interval was analytically notable (P< 0.000). The average effectiveness ratio (proportion of post operational UDVA and preoperational CDVA) was 1.36 ± 1.51 (extending from 0.2 to 8). Daxer A et al. showed the average UDVA developed by approximately 10 lines, from 0.07 LogMAR to 0.56 LogMAR, and the average CDVA developed by approximately 3 lines, from 0.42 LogMAR to 0.77 LogMAR [**6**].

UCVA improvement in Mahmood et al., Daxer et al. and Alio et al. studies were 7, 10 and 7 lines, respectively. With respect to CDVA, we observed an improve¬ment by 2 lines of LogMAR in 16 eyes (59%), which was compatible with the earlier research results [**5**,**6**-**11**]. 

It looks like that the MyoRing inserts have a higher possibility of myopic and astigmatic improvement in Keratoconus than ICRS apparently because of the more notable arc-shortening event achieved with a fully round mid-peripheral insert [**13**]. 

A notable center straightening was recognized after the operation, which was compatible with the produced refractive variation. The mean decrease in the average keratometry from the preoperative period to 6 months postoperatively was 6.41 ± 3.62 D. In Hosny et al. examined, the average variation in Km was 6.13 D (standard deviation, 4.37 D). This straightening event was similar to that described by (average difference in greatest keratometry of 9.60 D) after Ferrara loop portion implanta-tion in hard keratoconus [**16**]. It was also similar to those that Mahmood et al. described, Daxer et al., and Alio et al. who similarly applied the MyoRing in keratoconus [**5**-**11**].

The keratoconic patients were divided into 3 collections according to their preoperative keratometry (keratometry ≤48 D, 48-53 D, >53 D) and compared the outcomes between the 3 groups. In eyes with a preoperational keratometry of 48.0 D or smaller and of 48.0 to 53.0 D, the development in UDVA and CDVA and a decrease in SE and refractive cylinder were analytically notable. Good results were obtained in UDVA outcomes in all groups, but the development in CDVA was more significant in collection 1 (keratometry ≤48 D) compared to 2 other groups. Accordingly, it might be achieved that the MyoRing is a great choice for the subcollection of Keratoconus subjects. Furthermore, Alio et al. found a notable corneal flattening of an average amount of 8.03 diopters (D) [**11**] and a notable corneal flattening of an average amount of 9.78 D was found in Jabbarvand et al. study [**12**]. The mean K reading reduced by 5.76 D, from 48.96 D to 43.20 D in Daxer A study [**6**].

In our study, the average central corneal height was 422.42 ± 36.96 preoperatively and postoperatively it was 445.08 ± 28.00 (p= 0.020). Significant improvements were shown in the ocular awareness outcomes of all cases, there was no significant improvement between them for eyes with the central corneal thickness of less than 400 µm. Our finding was near to Jabbarvand et al. study that a meaningful rise in median corneal height [439.4 ± 19 to 452.2 ± 20 micrometers] was recognized while the 30 days post operational stage [**17**].

An intrastromal corneal loop was inserted in stromal depths of 300-μm by using FLT for all patients. Jabbarvand et al. assessed the clinical results of intrastromal MyoRing insertion at 2 various depths of 250 and 300 micrometers by using femtosecond laser. No variations were recognized in keratometry, visual and refractive results; in the 2 study groups. According to Jabbarvand et al. study, the conclusion was that an insertion depth of 250 micrometers has analogous results with the earlier employed 300 micrometers insertion depth and it may be suitable for the chosen subjects of keratoconus with smaller pachymetry [**18**].

In our study, no MyoRing was removed for side effects or complications but in Alio et al. study, the MyoRing explanation was finished in a very modern keratoconus because of the very poor visual result [**11**] and in Jabbarvand et al. study, MyoRing explanation was performed in (4%) 4 eyes, after a mechanical implantation of a MyoRing (Dioptex GmbH) [**12**].

## Conclusion

In conclusion, we discovered that the insertion of MyoRings by using FLT in cases of keratoconus notably decreased the spherocylindrical fault. We demonstrated a decrease in the average corneal keratometry and also spherical influence was extra notable than cylindrical influence decrease. As seen from the clinical data, this technique has the potential to correct notable myopic and astigmatic refractive faults. It appears that MyoRing insertion is a harmless and efficient method for the control of keratoconic cases. 

Further investigations with extended collections of patients, longer follow-up are needed to report more reliable outcomes with this implant.

**Acknowledgements**

The writers want to thank all the colleagues who supported the research, especially Shahla Rezaei for the assistance with the editing of the article.
